# Supplementary material for: Hidden duplicates: 10s or 100s of Indian trials, registered with ClinicalTrials.gov, have not been registered in India, as required by law
Source: PLoS One. 2020 Jun 19;15(6):e0234925. doi: 10.1371/journal.pone.0234925 (PMC7304601; doi:10.1371/journal.pone.0234925)
Supplement: S1 Data — (DOC) [file pone.0234925.s002.doc]

**S3 Legends. Legends for all the scripts used to process the data**

Section 1: Scraping Secondary id field from CTRI Database

Section 2: 1013 pairs of NCT-CTRI

Section 3: Scraping all the CTRI records

Section 4: Handling of missing values

Section 5: String Match Methodolgy

Section 6: Model for true-pair identification

Section 7: Identifying matches for the 1013 NCT records from amongst the 19,533 CTRI records, using string match

Section 8: Appling filters to the 2908 CTG records, which yielded 581 records.

Section 9: Finding unknown matches for 581 using text matching and the model.
