# Supplementary material for: Hidden duplicates: 10s or 100s of Indian trials, registered with ClinicalTrials.gov, have not been registered in India, as required by law
Source: PLoS One. 2020 Jun 19;15(6):e0234925. doi: 10.1371/journal.pone.0234925 (PMC7304601; doi:10.1371/journal.pone.0234925)
Supplement: S1 Text — An explanation of (a) what the coefficient of logistic regression implies, and (b) the model accuracy evaluation criteria. (DOC) [file pone.0234925.s003.doc]

**S7 Text. Details of the logistic regression model coefficient and model accuracy**

Below, we explain

(a) what the coefficient of logistic regression implies, and

(b) the model accuracy evaluation criteria.

A logistic regression model allows us to establish a relationship between a binary outcome variable and a group of predictor variables. The underlying algorithm of maximum likelihood estimation (MLE) determines the regression coefficient for the model that accurately predicts the probability of the binary dependent variable outcome. The algorithm stops when the convergence criterion is met or the maximum number of iterations are reached. Since the probability of any event lies between 0 and 1 (or 0% and 100%), when we plot the probability of dependent variable by independent factors, it will be a sigmoid curve.

Logit Transformation is defined as follows-

**Logit = Log (p/1-p) = log (probability of event happening/ probability of event not happening) = log (Odds)**

In logistic regression, we are only concerned about the probability of outcome dependent variable (success or failure).

What is p here? It turns out that p is the overall probability of being in honors class ( hon = 1 which is random in our case).

Further details are available in the S6 Table.

log(p/(1-p)) = logit(p) = intercept + coef.*Variable value

Another metric for classification of model is the confusion matrix.

The **confusion matrix** is a table test which is often used to describe the performance of the **classification model** on the test data for which the true values are already known, so we can use a **confusion matrix** to evaluate a model.


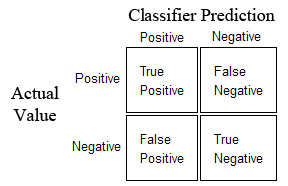


Below is the confusion matrix with 12 variables:


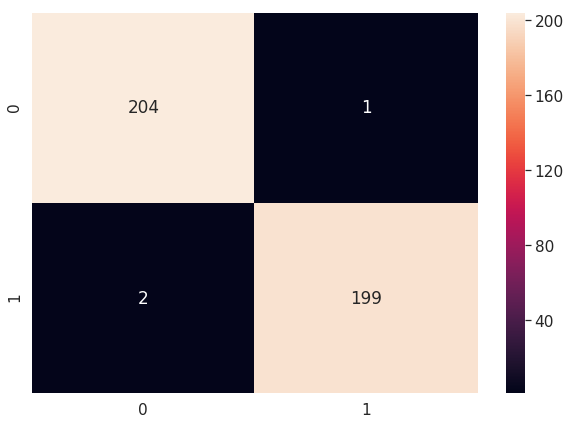


So, the model predicted true (0) when it was true (0) for 204 cases

predicted false (0) when it was true (1) for 1 cases

predicted true (0) when it was false (1) for 2 cases

predicted false (1) when it was false (1) for 199 cases

After removing the non-significant variables and model with variable: sponsor, title_scientific, title_public, condition, intervention, and primary outcome, we give below the confusion matrix.


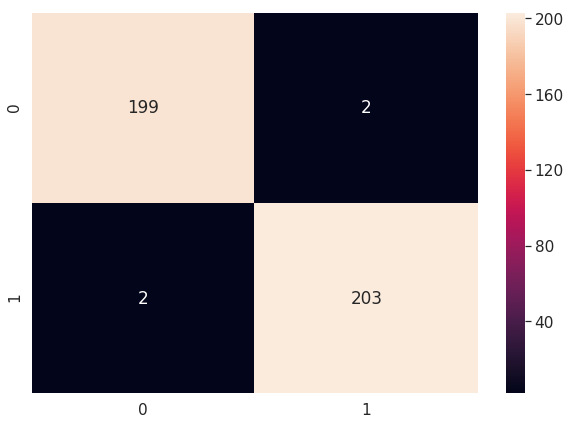


So, the model predicted true (0) when it was true (0) for 199 cases

predicted false (0) when it was true (1) for 2 cases

predicted true (0) when it was false (1) for 2 cases

predicted false (1) when it was false (1) for 203 cases

Thus, we can see that model prediction has not changed much, and we can get the same accuracy with fewer variables, which in-turn helps in avoiding the overfitting problem.

Thus, we decided to make predictions using a model with fewer variables.
