# Supplementary material for: Hidden duplicates: 10s or 100s of Indian trials, registered with ClinicalTrials.gov, have not been registered in India, as required by law
Source: PLoS One. 2020 Jun 19;15(6):e0234925. doi: 10.1371/journal.pone.0234925 (PMC7304601; doi:10.1371/journal.pone.0234925)
Supplement: Attachment — Submitted filename: RESPONSE TO REVIEWERS.doc [file pone.0234925.s013.doc]

**Comments to the Author**

1. Is the manuscript technically sound, and do the data support the conclusions?

The manuscript must describe a technically sound piece of scientific research with data that supports the conclusions. Experiments must have been conducted rigorously, with appropriate controls, replication, and sample sizes. The conclusions must be drawn appropriately based on the data presented.

Reviewer #1: Yes

Reviewer #2: Partly

4. Is the manuscript presented in an intelligible fashion and written in standard English?

PLOS ONE does not copyedit accepted manuscripts, so the language in submitted articles must be clear, correct, and unambiguous. Any typographical or grammatical errors should be corrected at revision, so please note any specific errors here.

Reviewer #1: Yes

Reviewer #2: No

 5. Review Comments to the Author

Please use the space provided to explain your answers to the questions above. You may also include additional comments for the author, including concerns about dual publication, research ethics, or publication ethics. (Please upload your review as an attachment if it exceeds 20,000 characters)

Reviewer #1: The aim of this study was to investigate whether all such trials registered with CTG, that included India as a location, also been registered with CTRI or not. The author reported three dozen of the 581 relevant CTG trials were not registered with CTRI.
The strength of this study was to investigate the theme extensively. However, there were some weakness in this study.

- - - - - - - - - - - - - - - - - - - - - - - - - - - - - - - - - - - - - - - - - -
First, the authors had better to add references to the following sentences in Introduction: “The biggest public repository is that of the United States of America (US), ClinicalTrials.gov (CTG), which holds over 320,000 records. The International Clinical Trials Registry Platform (ICTRP), established by the World Health Organization (WHO), enables users to search 18 open-access registries, including CTG. These registries cumulatively hold over 550,000 records.”

**Authors’ response: There were no references for these statements. We ascertained the numbers by visiting each website. We have now clarified this in the text, as follows:**

“The best known public repository is that of the United States of America (US), ClinicalTrials.gov (CTG). The International Clinical Trials Registry Platform (ICTRP), established by the World Health Organization (WHO), enables users to search 18 open-access registries, including CTG. We have visited the websites of each of these registries, and determined that they cumulatively hold 598,746 records, with CTG holding 335,963 (56%).”

- - - - - - - - - - - - - - - - - - - - - - - - - - - - - - - - - - - - - - - - - - - - - - - - -

Second, they had better to add references to the following recommendations in Conclusions: “Going forward, registries should make it compulsory to provide the Universal Trial Number. Alternatively, they should require trialists to provide the relevant ID if the study is registered elsewhere, or to confirm that it is not, and is unlikely to be, registered elsewhere. Further, the registry could coordinate with the drug regulator to compel trialists to provide this information, even retrospectively. If all registries enforce this, then it will go a long way in reducing the number of hidden duplicates, which inter alia will make it easier to identify whether or not the law has been broken in this manner.”

**Authors’ response: We have modified the para, as follows:**

“Going forward, registries should make it compulsory to provide the Universal Trial Number, as suggested by WHO’s ISCTR [International Standards for Clinical Trial Registries –Version 3.0. Geneva: World Health Organization; 2018. Licence: CCBY-NC-SA3.0IGO. Accessed 16 April 2020]. Alternatively, they should require trialists to provide the relevant ID if the study is registered elsewhere. If it is not, we suggest that trialists should explicitly confirm that the trial is not, and is unlikely to be, registered elsewhere. Further...”

- - - - - - - - - - - - - - - - - - - - - - - - - - - - - - - - - - - - - - - - - - - - - - - - -

Third, they had better to add description about penalties if the law about trial registration in India is broken.

**Authors’ response: Based on the other reviewer’s comment to make sub-headings more informative, we have renamed the Discussion section ‘The Year 2009’ to ‘India’s rule since mid-2009’. In this section, we have added the following:**

As mentioned above, since 15 June 2009 it has been mandatory to register regulatory trials running in India [Pandey et al. Challenges in Administering a Clinical Trials Registry:

Lessons from the Clinical Trials Registry-India; 2013. Pharm Med (2013) 27:83–93

DOI 10.1007/s40290-013-0009-3]. Should the responsible individual or organization fail to do so, the Licensing Authority is empowered to take actions such as issuing a warning letter, rejecting the results of the trial, or barring the investigator or sponsor from future trials for any length of time [Ministry of Health and Family Welfare. Govt of India. Notification. The Gazette of India: Extraordinary, Part II, Section 3, Subsection (i), New Delhi: 2019 Mar 19 [cited 2019 Apr 21], pgs 1-264. Available from: https://cdsco.gov.in/opencms/export/sites/CDSCO_WEB/Pdf-documents/NewDrugs_CTRules_2019.pdf].

- - - - - - - - - - - - - - - - - - - - - - - - - - - - - - - - - - - - - - - - - - - - - - - - -
Reviewer #2: The manuscript by Kumari et al. “Hidden duplicates: At least 3 dozen Indian trials, registered with ClinicalTrials.gov, have not been registered in India, as required by law” uses an informatics approach to identify clinical trials with India as location that are registered with the US federal register ClinicalTrials.gov (CTG in the manuscript) but not the Indian Clinical Trials Registry (CTRI). The manuscript is written in a rather convoluted way and the significance of the findings is not quite evident.

**Authors’ response:**

We have reworked the manuscript, and hope that it is less convoluted now. We have also expanded upon the significance, explained in another response below.

- - - - - - - - - - - - - - - - - - - - - - - - - - - - - - - - - - - - - - - - - - - - - - - - -

Major concerns
1) The authors describe exclusion of what they call “Phase 1” from their analysis (Page 22, Line 316) but then they say they have included “Early Phase 1” (Page 22, Line 319). The difference is not clear and should be further explained.

**Authors’ response:** In the original manuscript, we had stated “In order to identify trials for new drugs, we excluded those in phase 1, which may have included bioavailability and bioequivalence studies...”. We felt that BA/BE studies were unlikely to be called Early Phase 1 studies. In any case, there are only eight of such trials, out of 581, so their contribution to the results is minor.

We have added the line “BA/BE studies were unlikely to be called Early Phase 1 studies, and therefore we included the latter group of eight studies. ”.

- - - - - - - - - - - - - - - - - - - - - - - - - - - - - - - - - - - - - - - - - - - - - - - - -
2) The authors used 1) a training set of clinical trials for their algorithms and 2) several exclusion/inclusion criteria (e.g. studies posted to CTG after 2009) to come up with 581 studies registered in CTG with India as location and tried to determine the number so these trials that were also registered with CTRI. It is intriguing and worrisome that common hits from their two different algorithms were only 304 (52.3%). Although they claim “at least 3 dozen Indian trials” do not conform with the law, this number is not at all informative. Did they try to manually identify the 293 cases that are not common hits of their algorithms? They keep repeating in the discussion that 37-38 trials predicted to be registered only in CTG is a significant underestimate but no effort has been made to come up with a more precise number.

**Authors’ response:** We have now listed the 293 cases that are not common hits in Table 12. We have also manually checked a total of 50 pairs of them, to confirm that they are largely (47/50) incorrect matches.

In the initial manuscript, we were conservative in sticking to the figure of 37–38 hidden duplicates. We have now provided the entire range that the data reveals, that is roughly 50–300. We have also changed the title of the manuscript to reflect this.

We have expanded upon the significance, as follows: “Although 50–300 trials is a wide range, more important than the actual numbers is the fact of missing matches. The Indian regulator needs to launch an investigation, based on CTG records with India as a location, to determine whether particular sponsors have broken Indian law, and if so, to take necessary action.”

- - - - - - - - - - - - - - - - - - - - - - - - - - - - - - - - - - - - - - - - - - - - - - - - -
Minor concerns
1) Page 12, Lines 96-98: The sentence “57 records were common to the two searches, leaving 1013 unique trials that were registered in both databases (S1 Table)” should read “57 records were common to the two searches, leaving 1013 unique trials that were registered in either database (S1 Table)”.

**Authors’ response:** We have changed it.

- - - - - - - - - - - - - - - - - - - - - - - - - - - - - - - - - - - - - - - - - - - - - - - - -
2) Page 13, Line 114: Status should read “Recruitment status”

**Authors’ response:** In the Brief View of a record, and in the downloaded data of a given trial, it is just called status. Nevertheless, the full form is more informative, and we have changed it.

- - - - - - - - - - - - - - - - - - - - - - - - - - - - - - - - - - - - - - - - - - - - - - - - -

3) Several of the subheadings in the Results and Discussion sections are not informative (e.g. “The Year 2009”) and should be re-written.

**Authors’ response:** We have changed some of the sub-headings.

- - - - - - - - - - - - - - - - - - - - - - - - - - - - - - - - - - - - - - - - - - - - - - - - -

3) The bibliography is sloppily written and I would suggest the authors make sure it is presented in a uniform style. Additionally, I would suggest the following substitutions/changes/additions.
Page 10, Line 63: Add reference (Zarin DA, Tse T, Williams RJ, Carr S. Trial Reporting in ClinicalTrials.gov - The Final Rule. N Engl J Med. 2016;375(20):1998-2004. doi: 10.1056/NEJMsr1611785. PubMed PMID: WOS:000387856100015) after the sentence “In the US, it is mandatory that the results of most trials be deposited in CTG within one year of completion of the trial.”
Reference 6: Substitute reference (Turner L. ClinicalTrials.gov, stem cells and 'pay-to-participate' clinical studies. Regen Med. 2017;12(6):705-19. doi: 10.2217/rme-2017-0015. PubMed PMID: WOS:000417040900014) for the “Washington Post” article.
Reference 7: This is a book by the National Academies Press (https://www.nap.edu/catalog/12900/transforming-clinical-research-in-the-united-states-challenges-and-opportunities), cite appropriately.
Reference 10: Add complete reference.

**Authors’ response:** We have made these, and other, improvements to the list of References.

- - - - - - - - - - - - - - - - - - - - - - - - - - - - - - - - - - - - - - - - - - - - - - - - -
6. PLOS authors have the option to publish the peer review history of their article ([what does this mean?](https://journals.plos.org/plosone/s/editorial-and-peer-review-process#loc-peer-review-history)). If published, this will include your full peer review and any attached files.


If you choose “no”, your identity will remain anonymous but your review may still be made public.


**Do you want your identity to be public for this peer review?** For information about this choice, including consent withdrawal, please see our [Privacy Policy](https://www.plos.org/privacy-policy).

Reviewer #1: Yes: Masahiro Banno

Reviewer #2: No
