# Supplementary material for: Hidden duplicates: 10s or 100s of Indian trials, registered with ClinicalTrials.gov, have not been registered in India, as required by law
Source: PLoS One. 2020 Jun 19;15(6):e0234925. doi: 10.1371/journal.pone.0234925 (PMC7304601; doi:10.1371/journal.pone.0234925)
Supplement: Attachment — Submitted filename: Response to reviewers.doc [file pone.0234925.s014.doc]

For convenience, I include first our point-by-point response to Reviewer #1’s comments. By way of reference, your entire mail to us is appended after that.

6. Review Comments to the Author

Reviewer #1:

First, The authors had better to clarify when they visited the website in the following sentence of Introduction: “We have visited the websites of each of these registries, and determined that they cumulatively hold 598,746 records, with CTG holding 335,963 (56%).”

Authors’ response: We visited all the websites on 15 April 2020, and have modified the text accordingly.

- - - - - - - - - - - - - - - - - - - - - - - - - - - - - - - -

Second, they had better to add reference to the following sentence of Introduction: “We have visited the websites of each of these registries, and determined that they cumulatively hold 598,746 records, with CTG holding 335,963 (56%).” I consider the sentence need reference about the web site they visited. The example of reference is as follows: World Health Organization. International Clinical Trials Registry Platform (ICTRP): About the WHO ICTRP. 2019. Available at https://www.who.int/ictrp/about/en/. Accessed August 4, 2019.

Authors’ response: We have added the references for all 18 registries. Since a registry is slightly different from ICTRP, we consulted a document entitled ‘How to cite a record on a clinical trials register’ (<https://www.who.int/docs/default-source/documents/health-topics/how-to-cite.pdf?sfvrsn=d027a259_2>) to determine the format for referencing the registries.

- - - - - - - - - - - - - - - - - - - - - - - - - - - - - - - -
Third, they had better to add justifications or references about the following each sentence (one by one) in Conclusions: “Alternatively, they should require trialists to provide the relevant ID if the study is registered elsewhere. If it is not, we suggest that trialists should explicitly confirm that the trial is not, and is unlikely to be, registered elsewhere. Further, the registry could coordinate with the drug regulator to compel trialists to provide this information, even retrospectively. If all registries enforce this, then it will go a long way in reducing the number of hidden duplicates, which, inter alia, will make it easier to identify whether or not the law has been broken in this manner.” They had better to add explanation or references to connect Discussion with these recommendations in Conclusions. I think these recommendations in Conclusions appeared abruptly.

Authors’ response: We agree that the Conclusions appeared abruptly, and thank the reviewer for this comment. We have redone this section of the Conclusions (provided below), and it reads better now. However we were not able to provide references for two sentences, since they are our own suggestions (which we indicate or imply). We hope that the reviewer is happy with the flow now.

Original paragraph of the Conclusions:

Going forward, registries should make it compulsory to provide the Universal Trial Number, as suggested by WHO’s ISCTR [22]. Alternatively, they should require trialists to provide the relevant ID if the study is registered elsewhere. If it is not, we suggest that trialists should explicitly confirm that the trial is not, and is unlikely to be, registered elsewhere. Further, the registry could coordinate with the drug regulator to compel trialists to provide this information, even retrospectively. If all registries enforce this, then it will go a long way in reducing the number of hidden duplicates, which, inter alia, will make it easier to identify whether or not the law has been broken in this manner.

Re-written para:

In general, it is important to minimize the number of hidden duplicates in order to (a) reduce the bias both in meta-analyses of trial results that will inform clinical guidelines, and in any other analysis of large sets of registry records, and (b) ascertain whether the relevant Indian (or any other) law has been broken. Going forward, registries should make it compulsory to provide the Universal Trial Number, as suggested by WHO’s ISCTR [40]. Alternatively, and to reiterate the minimum standards recommended by ISCTR, they should require trialists to provide the relevant ID if the study is registered elsewhere. If it is not, ISCTR recommends that the secondary identifier field be filled with “Nil known”. We suggest that this requirement be taken one step further. Trialists should explicitly confirm that the trial is not, and is unlikely to be, registered elsewhere. Further, it should be possible for the registry to coordinate with the drug regulator to compel trialists to provide this information, even retrospectively. If all registries enforce such mechanisms to address this issue, then it will go a long way in reducing the number of hidden duplicates.

- - - - - - - - - - - - - - - - - - - - - - - - - - - - - - - -

-------- Forwarded Message --------

| Subject: | PLOS ONE Decision: Revision required [PONE-D-20-02492R1] - [EMID:deffcea6da95fb0b] |
| --- | --- |
| Date: | 2 Jun 2020 11:36:41 -0400 |
| From: | PLOS ONE <em@editorialmanager.com> |
| Reply-To: | PLOS ONE <plosone@plos.org> |
| To: | Gayatri Saberwal <gayatri@ibab.ac.in> |

PONE-D-20-02492R1
Hidden duplicates: 10s or 100s of Indian trials, registered with ClinicalTrials.gov, have not been registered in India, as required by law
PLOS ONE

Dear Dr. Saberwal,

Thank you for submitting your manuscript to PLOS ONE. After careful consideration, we feel that it has merit but does not fully meet PLOS ONE’s publication criteria as it currently stands. Therefore, we invite you to submit a revised version of the manuscript that addresses the points raised during the review process.

Please submit your revised manuscript by Jul 17 2020 11:59PM. If you will need more time than this to complete your revisions, please reply to this message or contact the journal office at plosone@plos.org. When you're ready to submit your revision, log on to https://www.editorialmanager.com/pone/ and select the 'Submissions Needing Revision' folder to locate your manuscript file.

Please include the following items when submitting your revised manuscript:

- A rebuttal letter that responds to each point raised by the academic editor and reviewer(s). You should upload this letter as a separate file labeled 'Response to Reviewers'.
- A marked-up copy of your manuscript that highlights changes made to the original version. You should upload this as a separate file labeled 'Revised Manuscript with Track Changes'.
- An unmarked version of your revised paper without tracked changes. You should upload this as a separate file labeled 'Manuscript'.

If you would like to make changes to your financial disclosure, please include your updated statement in your cover letter. Guidelines for resubmitting your figure files are available below the reviewer comments at the end of this letter.

If applicable, we recommend that you deposit your laboratory protocols in protocols.io to enhance the reproducibility of your results. Protocols.io assigns your protocol its own identifier (DOI) so that it can be cited independently in the future. For instructions see: [http://journals.plos.org/plosone/s/submission-guidelines#loc-laboratory-protocols](http://journals.plos.org/plosone/s/submission-guidelines#loc-laboratory-protocols)

We look forward to receiving your revised manuscript.

Kind regards,

Omid Beiki, M.D., Ph.D.
Academic Editor
PLOS ONE


[Note: HTML markup is below. Please do not edit.]

Reviewers' comments:

Reviewer's Responses to Questions

**Comments to the Author**

1. If the authors have adequately addressed your comments raised in a previous round of review and you feel that this manuscript is now acceptable for publication, you may indicate that here to bypass the “Comments to the Author” section, enter your conflict of interest statement in the “Confidential to Editor” section, and submit your "Accept" recommendation.

Reviewer #1: (No Response)

2. Is the manuscript technically sound, and do the data support the conclusions?

The manuscript must describe a technically sound piece of scientific research with data that supports the conclusions. Experiments must have been conducted rigorously, with appropriate controls, replication, and sample sizes. The conclusions must be drawn appropriately based on the data presented.

Reviewer #1: No

3. Has the statistical analysis been performed appropriately and rigorously?

Reviewer #1: Yes

4. Have the authors made all data underlying the findings in their manuscript fully available?

The [PLOS Data policy](http://www.plosone.org/static/policies.action#sharing) requires authors to make all data underlying the findings described in their manuscript fully available without restriction, with rare exception (please refer to the Data Availability Statement in the manuscript PDF file). The data should be provided as part of the manuscript or its supporting information, or deposited to a public repository. For example, in addition to summary statistics, the data points behind means, medians and variance measures should be available. If there are restrictions on publicly sharing data—e.g. participant privacy or use of data from a third party—those must be specified.

Reviewer #1: Yes

5. Is the manuscript presented in an intelligible fashion and written in standard English?

PLOS ONE does not copyedit accepted manuscripts, so the language in submitted articles must be clear, correct, and unambiguous. Any typographical or grammatical errors should be corrected at revision, so please note any specific errors here.

Reviewer #1: Yes

6. Review Comments to the Author

Please use the space provided to explain your answers to the questions above. You may also include additional comments for the author, including concerns about dual publication, research ethics, or publication ethics. (Please upload your review as an attachment if it exceeds 20,000 characters)

Reviewer #1: The authors revised the manuscript extensively. However, I have some comments.

First, The authors had better to clarify when they visited the website in the following sentence of Introduction: “We have visited the websites of each of these registries, and determined that they cumulatively hold 598,746 records, with CTG holding 335,963 (56%).”

Second, they had better to add reference to the following sentence of Introduction: “We have visited the websites of each of these registries, and determined that they cumulatively hold 598,746 records, with CTG holding 335,963 (56%).” I consider the sentence need reference about the web site they visited. The example of reference is as follows: World Health Organization. International Clinical Trials Registry Platform (ICTRP): About the WHO ICTRP. 2019. Available at https://www.who.int/ictrp/about/en/. Accessed August 4, 2019.

Third, they had better to add justifications or references about the following each sentence (one by one) in Conclusions: “Alternatively, they should require trialists to provide the relevant ID if the study is registered elsewhere. If it is not, we suggest that trialists should explicitly confirm that the trial is not, and is unlikely to be, registered elsewhere. Further, the registry could coordinate with the drug regulator to compel trialists to provide this information, even retrospectively. If all registries enforce this, then it will go a long way in reducing the number of hidden duplicates, which, inter alia, will make it easier to identify whether or not the law has been broken in this manner.” They had better to add explanation or references to connect Discussion with these recommendations in Conclusions. I think these recommendations in Conclusions appeared abruptly.

7. PLOS authors have the option to publish the peer review history of their article ([what does this mean?](https://journals.plos.org/plosone/s/editorial-and-peer-review-process#loc-peer-review-history)). If published, this will include your full peer review and any attached files.


If you choose “no”, your identity will remain anonymous but your review may still be made public.


**Do you want your identity to be public for this peer review?** For information about this choice, including consent withdrawal, please see our [Privacy Policy](https://www.plos.org/privacy-policy).

Reviewer #1: Yes: Masahiro Banno, MD, PhD


[NOTE: If reviewer comments were submitted as an attachment file, they will be attached to this email and accessible via the submission site. Please log into your account, locate the manuscript record, and check for the action link "View Attachments". If this link does not appear, there are no attachment files.]

While revising your submission, please upload your figure files to the Preflight Analysis and Conversion Engine (PACE) digital diagnostic tool, <https://pacev2.apexcovantage.com/>. PACE helps ensure that figures meet PLOS requirements. To use PACE, you must first register as a user. Registration is free. Then, login and navigate to the UPLOAD tab, where you will find detailed instructions on how to use the tool. If you encounter any issues or have any questions when using PACE, please email PLOS at [figures@plos.org](mailto:figures@plos.org). Please note that Supporting Information files do not need this step.

*In compliance with data protection regulations, you may request that we remove your personal registration details at any time.* [*(Remove my information/details)*](https://www.editorialmanager.com/pone/login.asp?a=r)*. Please contact the publication office if you have any questions.*
